# DRfit: a Java tool for the analysis of discrete data from multi-well plate assays

**DOI:** 10.1186/s12859-019-2891-5

**Published:** 2019-05-21

**Authors:** Andreas Hofmann, Sarah Preston, Megan Cross, H. M. P. Dilrukshi Herath, Anne Simon, Robin B. Gasser

**Affiliations:** 1 Griffith Institute for Drug Discovery, Griffith University, Nathan, Queensland 4111 Australia; 2 Department of Veterinary Biosciences, Melbourne Veterinary School, The University of Melbourne, Parkville, Victoria 3010 Australia; 3 Faculty of Science and Technology, Federation University, Ballarat, Victoria 3350 Australia; 4 Université Claude Bernard Lyon 1, Bâtiment Curien, Villeurbanne and Laboratoire Chimie et Biologie des Membranes et des Nanoobjets, Université de Bordeaux, Pessac, France

**Keywords:** Biochemical assays, Data analysis, Dose-response experiments, Drug discovery, Enzyme assays

## Abstract

**Background:**

Analyses of replicates in sets of discrete data, typically acquired in multi-well plate formats, is a recurring task in many contemporary areas in the Life Sciences. The availability of accessible cross-platform data analysis tools for such fundamental tasks in varied projects and environments is an important prerequisite to ensuring a reliable and timely turnaround as well as to provide practical analytical tools for student training.

**Results:**

We have developed an easy-to-use, interactive software tool for the analysis of multiple data sets comprising replicates of discrete bivariate data points. For each dataset, the software identifies the replicate data points from a defined matrix layout and calculates their means and standard errors. The averaged values are then automatically fitted using either a linear or a logistic dose response function.

**Conclusions:**

DRfit is a practical and convenient tool for the analysis of one or multiple sets of discrete data points acquired as replicates from multi-well plate assays. The design of the graphical user interface and the built-in analysis features make it a flexible and useful tool for a wide range of different assays.

**Electronic supplementary material:**

The online version of this article (10.1186/s12859-019-2891-5) contains supplementary material, which is available to authorized users.

## Background

Contemporary research in the Life Sciences makes use of many experimental techniques that acquire data from multi-well plate assays. As intended by the manufacturers of plate readers, the acquisition software provided with the equipment can be used to process and analyse data generated by individual instruments. In particular, in busy laboratory environments, off-line analysis is, in our experience, the preferred option of many users; however, in most instances, this requires the purchase of software licences. Additionally, most of the commercially available software in this context is available only for Microsoft Windows operating systems, such as, for example, MultiCycle (ActiMetrics), Gen5 (BioTek), Multi-Mode Analysis Software (Molecular Devices), and Multiwell-Analyzer (Multi Channel Systems).

Few non-commercial software applications addressing multi-well plate data analysis have been reported. The majority of them have been specifically designed for particular tasks, such as the analysis of single-cell migration [1] or differential scanning fluorimetry data [2]. The application BiAnaCa was reported as a tool for general biochemical assays [3], but does not appear to be available for download. The open source laboratory information system Brunn [4] embraces an end-to-end concept for multi-well plate experiments and has been implemented as a database system requiring the setup of server and clients. In addition to the mentioned applications, which all provide a graphical user interface, there are also libraries for the statistical package R [5], such as ‘platetools’ (https://cran.r-project.org/package=platetools), ‘plater’ (https://cran.r-project.org/package=plater) and ‘phenoScreen’ (https://rpubs.com/Swarchal/phenoScreen); however, neither of them have any dose-response fitting functionality.

Based on the concept of a program collection for structural biology and biophysical chemistry (PCSB) [6], we have developed several stand-alone portable applications for a variety of analytical tasks with an emphasis on intuitive and convenient usage. Here, we present a user-friendly application, called DRfit, for the analysis, visualisation and curation of bivariate discrete data obtained from multi-well plate assays. The software includes automated and manual fitting of averaged data from replicate data sets as well as convenient data import and export features.

DRfit closes a current gap in the landscape of multi-well plate analysis software and provides a free cross-platform tool for interactive analysis of multi-well plate data for a wide variety of assays. Due to multi-language support, the intuitive user interface as well as implemented features, the software should appeal to students in tertiary education and academic laboratories alike.

### Implementation

#### Design

DRfit has been designed as an analysis pipeline to process replicate data obtained from end-point assays (discrete data) in a defined layout. In most cases, such data will be acquired in assays using multi-well plates, but it is also possible to enter data from individual experiments. Based on a user-specified plate setup, replicate data can be processed for multiple datasets. For each data set, the mean and variation (standard deviation, standard error, 95% confidence interval) will be calculated and an automated fit to a user-selected function will be attempted. Numerical results of the automated fitting are displayed in a spreadsheet. With a mouse right-click on a row in this spreadsheet, the user can trigger a plot window to visualise the averaged data points in a scatter plot with the superimposed fit. From the fitting panel in this window, the user can trigger new data fits by entering start values for the fit function. Additionally, one can adjust the current fit parameters manually through the use of graphical sliders.

In the design of this software, special emphasis has been placed on an intuitive and clean graphical user interface (GUI) that makes this software easy to use. The GUI is available in English, French and German. Data can be entered directly into the DRfit spreadsheets or imported from Microsoft Excel files. To allow tracking of calculations and data transformation, ASCII log files are output at every step throughout the analysis pipeline. For user convenience, data plots and superimposed fits can be written automatically into image files and all results from an analysis session can be exported at once into a Microsoft Excel workbook.

#### Architecture

This software builds on the Java libraries that we have developed for the Program Collection for Structural Biology and Biophysical Chemistry [6]; in particular, it uses the GraphPanel class which was originally developed for the interactive analysis and fitting application SDAR [7]. For automated fitting, the Levenberg-Marquardt minimisation module developed by J. P. Lewis (http://scribblethink.org/index.html) has been implemented; import/export functionality of Microsoft Excel-formatted spreadsheets uses JExcelApi established by Andy Khan (http://jexcelapi.sourceforge.net/). DRfit is platform-independent and can be deployed on Windows, Linux or MacOS; a detailed software manual (see Additional file 1), example data sets (see Additional file 2), as well as a video tutorial are available on the project web site.

### General concept

**Fig. 1:**
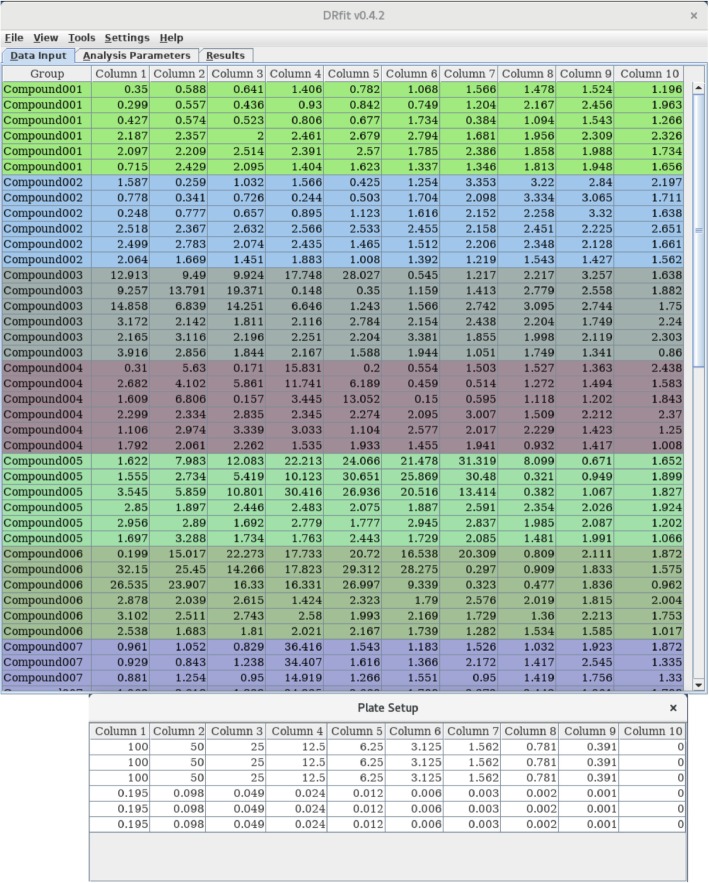
View of the *Data Input* and *Plate Setup* spreadsheets. Replicate data for multiple data sets (‘groups’) are entered into the *Data Input* panel; individual groups are highlighted by different background colours. The groups need to adhere to the same data layout as defined in the *Plate Setup* spreadsheet using the numerical values of the individual ‘tests’ as labels

Discrete data of replicates of experiments for a particular ‘group’ (e.g., an enzyme, compound, etc) under varying conditions (‘tests’; e.g., concentration of a substrate or ligand) need to be organised as a matrix in the *Plate Setup* spreadsheet using the numerical values of the individual ‘tests’ as labels; these ‘test’ values constitute the abscissa in the data plots. The observed values of the individual experiments are entered in the *Data Input* spreadsheet; the values of the observable constitute the ordinate in the data plots. Data for both the plate setup and the observables for the different ‘groups’ can be imported from spreadsheet files in Microsoft Excel (1997–2002) format or entered manually into the DRfit spreadsheets. In the *Data Input* spreadsheet, individual ‘groups’ are highlighted by different background colours (see Fig. 1).

**Fig. 2:**
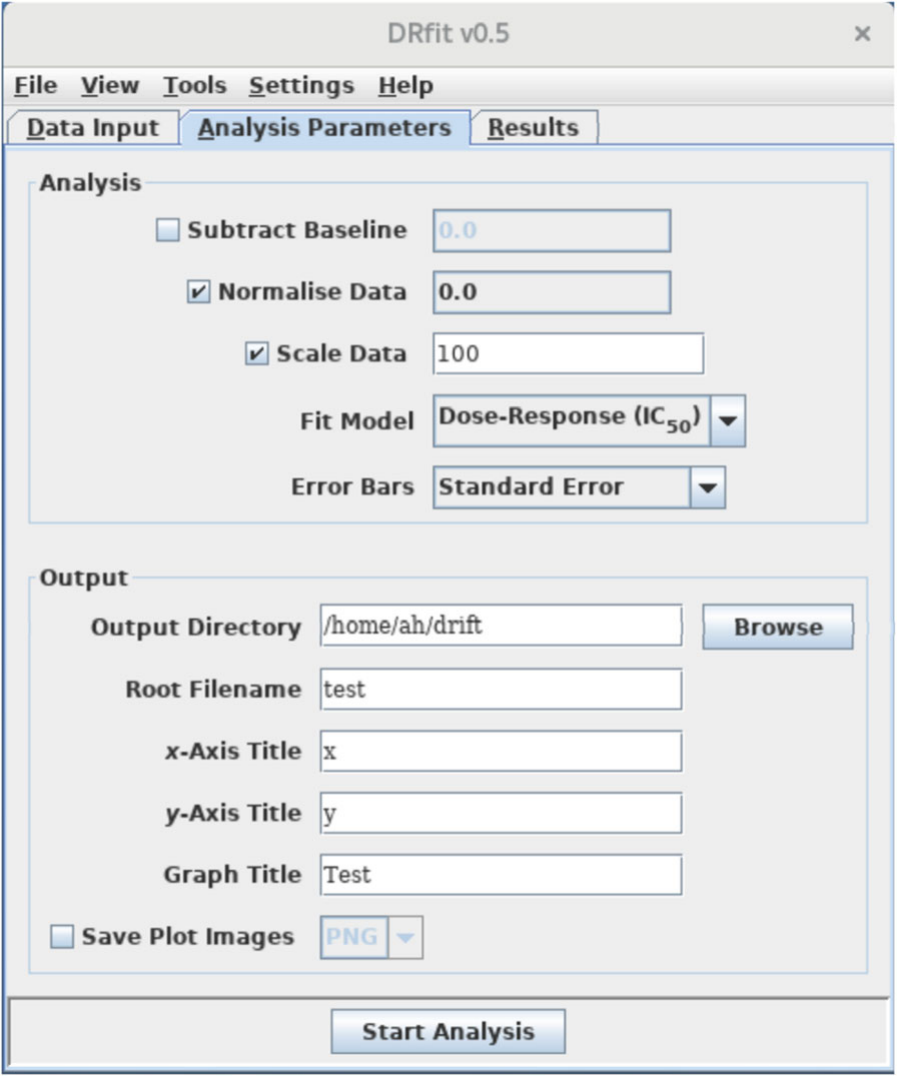
View of the panel for input of analysis parameters. For baseline subtraction and data normalisation, experimental replicate sets can be selected from drop down lists, identified by the numerical labels used in the *Plate Setup* spreadsheet. The analysis pipeline is started by clicking the *Start Analysis* button. Fitting results are accumulated in the *Results* panel from which data plots can be triggered for visual inspection

Parameters for processing of the data are set in the panel *Analysis Parameters* (see Fig. 2). The drop-down menus labelled *Subtract Baseline* and *Normalise Data* are populated with the unique values of cells from the *Plate Setup* window and allow for baseline subtraction and data normalisation (in this order). If *Scale Data* is activated, the user can specify a numerical value that is multiplied with all data values; this step is applied after normalisation and before any further data analysis. From the *Fit Model* drop down menu, a logistic dose response model and linear model can be chosen.

Further processing parameters include directory settings and output file names, titles for the *x*- and *y*-axis as well as an overall title for the graphs. Image files with scatter plots and superimposed fits can be generated automatically in either PNG, SVG or TIFF format; the resolution as well as width and height of the generated image files can be modified *Miscellaneous Settings* from the menu bar.

**Fig. 3:**
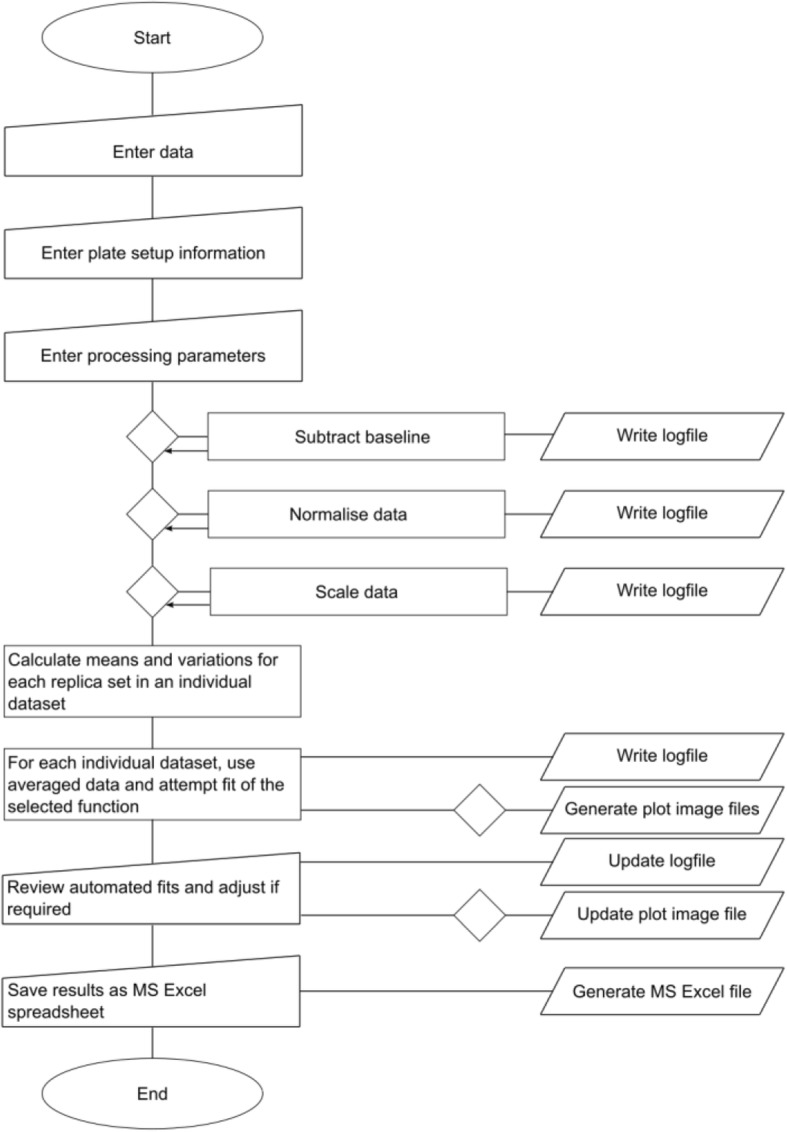
Flow diagram. The flow diagram summarises the major individual steps of the data analysis pipeline implemented in DRfit

An overview of the processing pipeline with the individual steps is shown in the flow diagram in Fig. 3.

### Outliers

Frequently, series of data points obtained from experimental measurements include individual observations that are much smaller or larger then reasonably expected. Such ‘outliers’ can be thought of as data points that are ‘far away’ from the rest of the data. When suspecting that a data point might be an outlier, it is common to perform the statistical analyses twice, once with the suspected outlier and once without it. Therefore, we have included features into DRfit that enable the user to remove suspected outliers, either manually or in an automated fashion.

Individual cells or cell selections in the *Data Input* spreadsheet can be marked as outliers via a popup menu that is obtained with a right mouse click. Outliers will not be taken into account when calculating the means and variations. If all replicates of a data point are marked as outliers, then this data point will become masked in the plot and be ignored for fitting. Alternatively, outliers can be identified in the data plots shown in the popup window for a ‘group’ in the *Results* spreadsheet. By drawing a rectangular selection with a mouse left-drag around one or more data points, all replicates of the selected ‘test’ become masked in the plot and are identified as outliers in the *Data Input* spreadsheet.

In addition to manual outlier selection, it is possible to subject all data in the *Data Input* spreadsheet to an automatic test for outliers within replicate sets. Numerical approaches to dealing with outliers employ either weighted linear regression or statistical tests developed for identifying outliers amongst replicate values, such as the Grubbs test or Dixon’s Quotient (Q) test [8]. The so-called 3σ edit rule, based on the fact that the probability to observe data points further away from the mean than 3 standard deviations is only 0.3% [9], has proven rather ineffective in practical applications [10]. Owing to a recommendation by the International Organization for Standardization, Dixon’s Q test has largely been replaced with the Grubbs test as a commonly used procedure [11]. This latter test is based on the difference of the mean of the sample and the farthermost data, and also considers the standard deviation [12, 13]. Capitalising on Java classes developed earlier for our DMAN software [2], we have implemented Grubbs’ test for outliers in the present software. This can be actioned through *Check for Outliers* from the *Tools* menu bar item. If this feature is activated, an outlier test will be done as the very first step of the automated analysis pipeline.

### Fit results

Results of the automated fitting for each ‘group’ are summarised in the spreadsheet of the *Results* panel. A mouse right-click on a row in the *Results* spreadsheet opens a window that shows the plot of means (circles) and the chosen variation parameter (error bars) for this data set, as well as the fitted function if successful. The values of the fitted parameters are displayed in the text fields on the right hand side of the plot. Changing any of those values will adjust the fitted curve; the goodness of fit statistics are also updated. It is also possible to adjust the fitted curve by moving any of the sliders provided for each of the fit parameters. Automated fitting of the data can be actioned by clicking the *New Fit* button; in this case, the values currently displayed for each of the fit parameters will be taken as guess parameters of the auto-fitting procedure.

## Results

### Application example: dose response assays

Many studies are concerned with dose response or receptor-ligand binding assays. In particular in drug discovery applications, increasing concentrations of small-molecule compounds are tested in protein-based or whole organism assays. Most frequently, such data are analysed using a four-parameter logistic equation that results in a sigmoid curve, in which the point of inflection yields the widely used parameter IC_50_/EC_50_. Previously, we reported a low-cost nematode motility assay suitable for the screening of compounds for anthelmintic activity [14, 15]. In our ongoing efforts to ‘streamline’ data analysis for this motility assay, DRfit has been successfully deployed, leading to further improvements of time required to conduct screening studies. Example data sets (see Additional file 2) and a video tutorial showing a step-by-step dose response analysis are available from the project website.

### Application example: enzyme kinetics

Most commonly, enzyme kinetics are analysed using the initial rate. From endpoint assays, a time-dependent plot of substrate/product concentration can be produced. Typically, the initial rate is obtained from tangents fitted to the steady state curves in the origin, since their slopes yield the initial rate [16]. Using the linear fit model of DRfit, initial rate analysis of enzyme kinetics data, acquired in a multi-well format, can be carried out conveniently. We have used DRfit to assess the standard (= non burst-like) steady-state kinetics of trehalose-6-phosphate phosphatases [17].

## Conclusions

DRfit is a cross-platform software tool for processing multiple sets of discrete data points acquired as replicates. All data sets to be processed within one session need to possess the same layout. This current software has been designed with practicality and convenience in mind and features a range of processing options, including automated fitting and generation of image files as well as import/export functionality from/to Microsoft Excel spreadsheets.

## Additional files

Additional file 1: Software manual. (PDF 267 kb)

Additional file 2: Example session file. (DRFIT 46 kb)

## Abbreviations

GUI: Graphical user interface
