# Supplementary material for: DRfit: a Java tool for the analysis of discrete data from multi-well plate assays
Source: BMC Bioinformatics. 2019 May 21;20:262. doi: 10.1186/s12859-019-2891-5 (PMC6528253; doi:10.1186/s12859-019-2891-5)
Supplement: Supplementary file 1 — Software manual. (PDF 267 kb) [file 12859_2019_2891_MOESM1_ESM.pdf]

# DRfit

# A Java tool for analysis of discrete multi-well plate data

## version 0.5

# Table of Contents


**1. SUMMARY....3**

**2. GENERAL....3**

2.1 Starting the software....3
2.2 Starting the software from terminal....3

**3. GENERAL CONCEPT....4**

3.1 Flow diagram....5

**4. STEP-BY-STEP DESCRIPTION OF THE ANALYSIS PIPELINE AND GUI....6**

4.1 Enter the multi-well plate setup....6
4.2 Enter the data to be processed....6
4.3 Mark outliers (optional)....6
4.4 Enter the processing parameters....6
4.5 Start the data analysis....7
4.6 Results....7

**5. FIT MODELS....7**

**6. STATISTICAL PARAMETERS....8**

**7. NON-PCSB JAVA LIBRARIES USED IN THIS PROGRAM....8**

**8. REFERENCES....8**

**9. APPENDIX 1: COPYRIGHT, LICENSE AND DISCLAIMER....8**

## 1. Summary

DRfit is a Java tool for the analysis of multiple data sets comprising replicates of discrete bivariate data. In particular, the software has been designed to allow convenient analysis of data structures obtained from multi-well plate assays. Multiple different data sets collected with the same plate layout can be processed at once.
The organisation of data points on the multi-well plate as well as their values in individual data sets can be entered by the user into a built-in spreadsheet or loaded directly from a spreadsheet in Microsoft Excel format. The analysis pipeline can be configured to subtract background, normalise and scale data. Outliers within sets of replicate data points can be identified automatically using a Grubbs test but also marked up manually by the user.
For each data set, the software identifies the replicate data points from the defined matrix (plate) layout and calculates their means and standard errors. The averaged values are then automatically fitted using either a linear or a logistic dose response function. All fits can be visualised and interactively amended by the user. Data plots with fits can be written automatically for each data set and all results from the data analysis can be written into an Excel-formatted spreadsheet.
Please cite [1] when using this software.

## 2. General

This software is part of the Program Collection for Structural Biology and Biophysical Chemistry (PCSB) [1]; non-PCSB libraries used by this software are listed in section 7.

DRfit has been compiled using Sun/Oracle Java 1.7 and tested using Java versions 1.7 and 1.8.

### 2.1 Starting the software

To run stand-alone Java applications on Windows and Macintosh OS, simply double-click the `.jar` file.

Under Linux, depending on the Java installation, the software might run by typing

```
java -jar {drfit-archive}.jar
```

### 2.2 Starting the software from terminal

A few options are available when starting DRfit from the terminal.

Start the GUI from the terminal:

```
java -jar {drfit-archive}.jar
```

Start the GUI and open a DRfit session file:

```
java -jar {drfit-archive}.jar {data-file}.drfit
```

Start the GUI with command line options:

```
java -jar {drfit-archive}.jar [options]
```

Options:

| | |
|---|---|
| `-debug` | be verbose |

### 3. General concept

This software has been designed as an analysis pipeline that calculates means and standard errors of discrete replicate data for different individual data sets (`group`s) collected in multi-well plate format. Individual data sets can be chosen as background (subtracted from all data points) or reference for normalisation. It is also possible to apply a scaling factor to all data.

The averaged data for each `group` are then fitted to either a linear or logistic dose response function. For each `group`, the results of the automated fits are tabulated in a spreadsheet, from which the user can activate a plot window showing the mean values and standard errors as well as the fitted function. From the plot window, fits can be repeated with manually adjusted guess values for the fit parameters; alternatively, the fits can be manually adjusted.

Log files (ASCII files in CSV format with space separation) containing the data at each step throughout the analysis are automatically written into the user-specified directory. Data plots can also automatically be generated in either PNG, SVG or TIFF format. Both log files and image files are automatically updated when the user manually triggers re-fitting of a data set.

After a full analysis has been carried out, all data (including input and results) can be written into spreadsheet file in Microsoft Excel (1997-2003) format (`*.xls`). A DRfit status file (`*.drfit`) can also be written and thus allows resuming of the analysis session at a later time.

The GUI is available in English, French and German.

### 3.1 Flow diagram

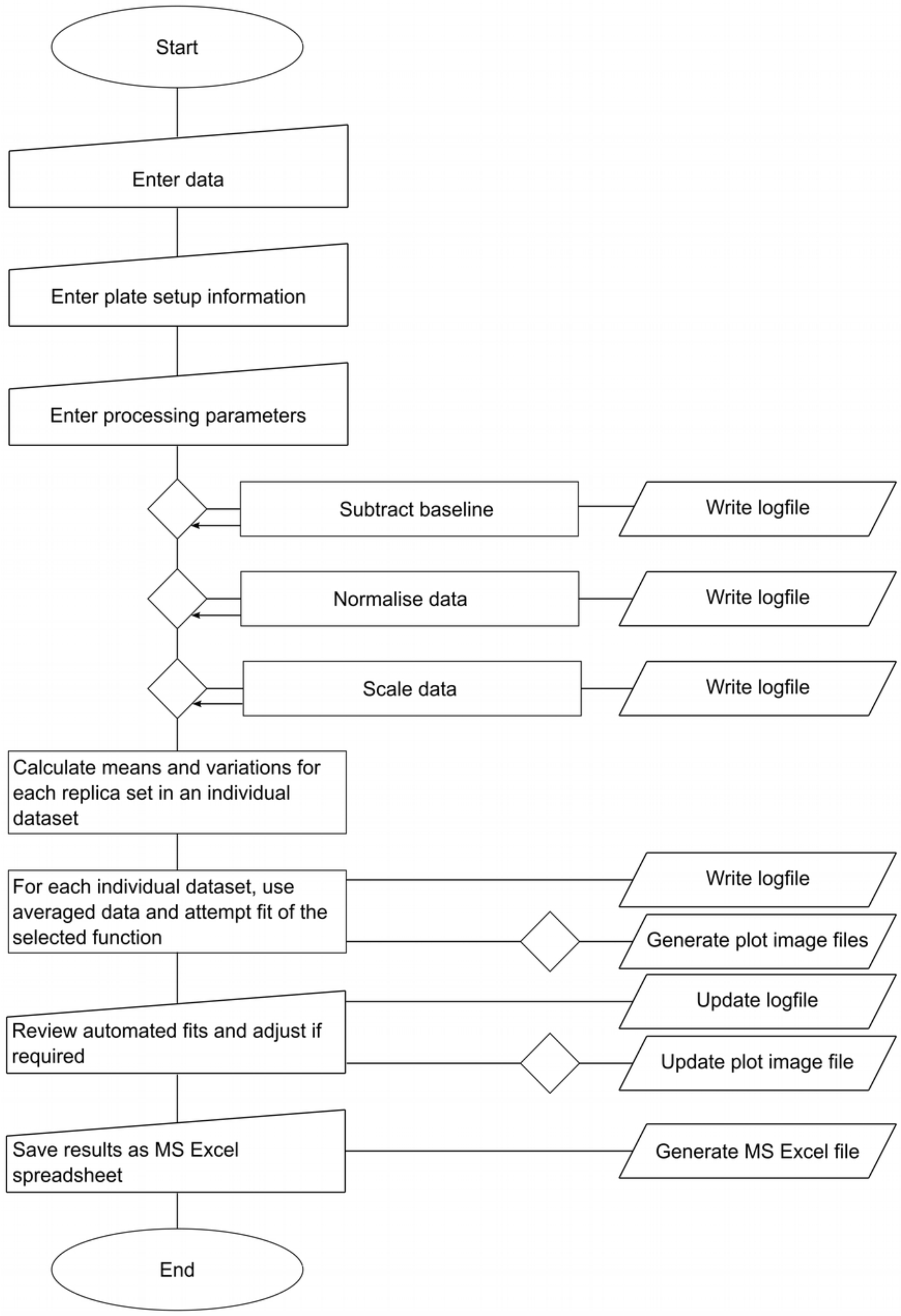

# 4. Step-by-step description of the analysis pipeline and GUI

## 4.1 Enter the multi-well plate setup

Information about the setup of the multi-well plate is entered in the spreadsheet of the **Plate Setup** window. Data can be entered manually into the cells, copy-pasted through the clipboard or loaded from a spreadsheet file in MS Excel (1997-2002) format. A **mouse right-click** in a cell opens a popup menu that offers features to insert and remove columns and rows. When copy-pasting data through the clipboard or loading an external file with plate setup information, cells are automatically added to the spreadsheet if required. A plate information file can be loaded through the menu bar using **File – Import Plate Setup**. The **Plate Setup** window can be hidden and displayed using the menu feature **View – Plate Setup**.

Data in the **Plate Setup** spreadsheet should all be numerical as their values constitute the *x*-axis values of the plot to be generated. The only exception are wells which hold data to be used for background subtraction; these wells may contain non-numerical characters (e.g. 'baseline', etc).

Single replicate data can be processed by defining the plate layout as a $n \times 1$ matrix ($n$ rows, 1 column). Importantly, the plate layout does not have to match standard plate dimensions (e.g. 8 rows, 12 columns). If, for instance, only 3 rows and 5 columns are used on a standard 8×12 plate, then only a 3×5 plate layout matrix needs to be provided.

## 4.2 Enter the data to be processed

The data to be processed are entered in the spreadsheet under panel tabbed **Data Input**. The first column ('**Group**') identifies the current row as a member of an individual data set. All data to be processed need to be numerical and given in the following columns, following the layout defined in the **Plate Setup** window. For each data set, there need to be as many contiguous rows in the **Data Input** spreadsheet as there are rows in the **Plate Setup** spreadsheet. The columns labelled 'Column 1', Column 2', … need to hold numerical data only; there need to be as many columns in addition to the **Group** column in the **Data Input** spreadsheet as there are columns in the **Plate Setup** window.

Data can be entered manually into the cells, copy-pasted through the clipboard or loaded from a spreadsheet file in MS Excel (1997-2002) format. A **mouse right-click** in a cell opens a popup menu that offers features to insert and remove columns and rows. When copy-pasting data through the clipboard or loading an external file with data, cells are automatically added to the spreadsheet if required. A data file can be loaded through the menu bar using **File – Import Data**.

Many different datasets that follow the defined plate layout can be processed at once.

## 4.3 Mark outliers (optional)

Once all data have been entered, it is possible to mark individual cells or cell selections in the **Data Input** spreadsheet as outliers. Outliers will not be taken into account when calculating the means and variations. If all replicates of a data point are marked as outliers, then this data point will become masked in the plot and be ignored for fitting.

In order to manually mark cells as outliers, use a **mouse right-click** and select **Mark as outlier** from the popup menu. Alternatively, outliers can identified in the data plots shown in the popup window for a **Group** in the **Results** spreadsheet. By drawing a rectangular selection with a **mouse left-drag** around one or more data points, all replicates of the selected data point become masked in the plot and are identified as outliers in the **Data Input** spreadsheet.

All data can also be subjected to an automatic test for outliers within replicate sets using a Grubbs test [2,3]. This can be actioned through the menu item **Tools – Check for Outliers**.

### 4.4 Enter the processing parameters

Processing parameters are entered in the panel tabbed **Analysis Parameters**. The drop-down menus labelled **Subtract Baseline** and **Normalise Data** are populated with the unique values of cells from the **Plate Setup** window. If **Subtract Baseline** is activated, the values of the replicate data points of the selected wells will be averaged and subtracted from all data prior to any further analysis. If **Normalise Data** is activated, the values of the replicate data points of the selected wells will be averaged and used as divisors for all data; this step is applied after baseline subtraction and before any further data analysis. If **Scala Data** is activated, the user can specify a numerical value that is multiplied with all data values; this step is applied after normalisation and before any further data analysis. The **Fit Model** drop down menu allows choosing the mathematical equation to be fitted to the averaged data. At the moment, a logistic dose response model and linear model have been implemented. The statistical parameter used for the error bars can be selected from the **Error Bars** drop down menu.

In the text field **Output Directory** the user needs to specify the directory into which the output files will be written; all files will start with the name given as **Root Filename**. The titles of the plot axes can be amended in the text fields ***x*-Axis Title** and ***y*-Axis Title**. All data plots will be titled using the **Group** name; any text to be added as a pre-fix can be entered under **Graph Title**. If **Save Plot Images** is activated, an image file (PNG, SVG or TIFF format) will automatically be generated in the requested format and written to the output directory. The resolution as well as width and height of the generated image files can be modified through the menu bar using **Settings – Miscellaneous**.

### 4.5 Start the data analysis

The data analysis pipeline is started by clicking the button **Start Analysis** in the **Analysis Parameters** panel. Data from the **Data Input** panel will be read and processed using the set parameters; replicate sets for each individual data set will be assembled using the information from the **Plate Setup** window.

When loading a previously saved status file, an analysis run with **Start Analysis** is required in order to be able to write out all results into an MS Excel spreadsheet.

### 4.6 Results

For each step, a CSV-formatted ASCII file is generated. All files are accumulated in the directory specified as **Output Directory** and file names start with the name given as **Root Filename**.

Results of the automated fitting for each data set are summarised in the spreadsheet of the **Results** panel. A **mouse right-click** on a row in the Results spreadsheet opens a window that shows the plot of means (circles) and chosen variation parameter (error bars) for this data set, as well as the fitted function if successful. The values of the fitted parameters are displayed in the text fields on the right hand side of the plot. Changing any of those values and pressing **Enter** will adjust the fitted curve; the goodness of fit statistics are also updated. It is also possible to adjust the fitted curve by moving any of the sliders provided for each of the fit parameters. Automated fitting of the data can be actioned by clicking the **New Fit** button; in this case, the values currently displayed for each of the fit parameters will be taken as guess parameters of the auto-fitting procedure. Clicking **Cancel** dismisses the popup window without changes. Clicking **OK** commits the changes and the Results spreadsheet, log files and plot image files will automatically be updated.

## 5. Fit models

The following fit models are currently implemented:

| | | |
|---|---|---|
| Dose Response ($IC_{50}$) | $y = \text{Top} + \dfrac{\text{Bottom} - \text{Top}}{1 + \left(\dfrac{x}{IC_{50}}\right)^{-\text{Slope}}}$ | This will fit a logistic four-parameter equation. The automated fitting routine uses a Levenberg-Marquardt minimisation. |
| Linear | $y = m \cdot x + t$ | This will fit a linear equation. The automated fitting routine uses simple linear regression. |

## 6. Statistical parameters

Fit statistics are calculated to assess the goodness of fit between the fitted function and the discrete data. The following parameters are calculated considering all non-masked data points:

| | |
|---|---|
| $R\text{-factor} = \dfrac{\sum \lvert y_{exp} \rvert - \lvert y_{fit} \rvert}{\sum \lvert y_{exp} \rvert}$ | A perfect fit has an *R*-factor of 0. |
| $R^2 = \dfrac{\sum (y_{fit} - \mu_{exp})^2}{\sum (y_{exp} - \mu_{exp})^2}$ with the mean $\mu_{exp} = \dfrac{1}{n} \cdot \sum y_{exp}$ | A perfect fit has an $R^2$ parameter of 1. |

## 7. Non-PCSB Java libraries used in this program

DRfit makes use of the following Java libraries not authored by us:

- Apache™ Batik SVG toolkit; http://xmlgraphics.apache.org/batik/index.html
- Apache™ XML Graphics; https://xmlgraphics.apache.org/
- JExcelApi by Andy Khan; http://jexcelapi.sourceforge.net/
- Levenberg-Marquardt in Java by J. P. Lewis; http://scribblethink.org/index.html
- Simple API For CSS by World Wide Web Consortium; https://mvnrepository.com/artifact/org.w3c.css/sac
- W3C DOM SVG by World Wide Web Consortium; https://mvnrepository.com/artifact/org.w3c.dom/org.w3c.dom.svg

## 8. References

1 Hofmann A & Wlodawer A (2002) PCSB--a program collection for structural biology and biophysical chemistry. *Bioinformatics* **18**, 209–210.
2 Grubbs F (1969) Procedures for detecting outlying observations in samples. *Technometrics* **11**, 1–21.
3 Stefansky W (1972) Rejecting outliers in factorial designs. *Technometrics* **14**, 469–479.

## 9. Appendix 1: Copyright, License and Disclaimer

Copyright © 1999-2019
Hofmann Laboratory, http://www.structuralchemistry.org/pcsb/

License
This program is free software; you can redistribute it and/or modify it under the terms of the GNU Affero General Public License version 3 as published by the Free Software Foundation. You should have received a copy of the GNU Affero General Public License along with this program (file COPYING); if not, see http://www.gnu.org/licenses or write to the Free Software Foundation, Inc., 51 Franklin Street, Fifth Floor, Boston, MA, 02110-1301 USA.

Disclaimer
This program is distributed in the hope that it will be useful, but without any warranty; without even the implied warranty of merchantibility or fitness for a particular purpose. See the GNU Affero General Public License for more details.
